# Ploidy Reductions in Murine Fusion-Derived Hepatocytes

**DOI:** 10.1371/journal.pgen.1000385

**Published:** 2009-02-20

**Authors:** Andrew W. Duncan, Raymond D. Hickey, Nicole K. Paulk, Andrew J. Culberson, Susan B. Olson, Milton J. Finegold, Markus Grompe

**Affiliations:** 1 Oregon Stem Cell Center, Oregon Health and Science University, Portland, Oregon, United States of America; 2 Department of Molecular and Medical Genetics, Oregon Health and Science University, Portland, Oregon, United States of America; 3 Department of Pathology, Texas Children's Hospital, Houston, Texas, United States of America; 4 Papé Family Research Institute, Department of Pediatrics, Oregon Health and Science University, Portland, Oregon, United States of America; National Institute of Diabetes and Digestive and Kidney Diseases, United States of America

## Abstract

We previously showed that fusion between hepatocytes lacking a crucial liver enzyme, fumarylacetoacetate hydrolase (FAH), and wild-type blood cells resulted in hepatocyte reprogramming. FAH expression was restored in hybrid hepatocytes and, upon *in vivo* expansion, ameliorated the effects of FAH deficiency. Here, we show that fusion-derived polyploid hepatocytes can undergo ploidy reductions to generate daughter cells with one-half chromosomal content. Fusion hybrids are, by definition, at least tetraploid. We demonstrate reduction to diploid chromosome content by multiple methods. First, cytogenetic analysis of fusion-derived hepatocytes reveals a population of diploid cells. Secondly, we demonstrate marker segregation using ß-galactosidase and the Y-chromosome. Approximately 2–5% of fusion-derived FAH-positive nodules were negative for one or more markers, as expected during ploidy reduction. Next, using a reporter system in which ß-galactosidase is expressed exclusively in fusion-derived hepatocytes, we identify a subpopulation of diploid cells expressing ß-galactosidase and FAH. Finally, we track marker segregation specifically in fusion-derived hepatocytes with diploid DNA content. Hemizygous markers were lost by ≥50% of *Fah*-positive cells. Since fusion-derived hepatocytes are minimally tetraploid, the existence of diploid hepatocytes demonstrates that fusion-derived cells can undergo ploidy reduction. Moreover, the high degree of marker loss in diploid daughter cells suggests that chromosomes/markers are lost in a non-random fashion. Thus, we propose that ploidy reductions lead to the generation of genetically diverse daughter cells with about 50% reduction in nuclear content. The generation of such daughter cells increases liver diversity, which may increase the likelihood of oncogenesis.

## Author Summary

The liver comprises many different types of cells, including hepatocytes. Hepatocytes perform numerous physiological functions, such as detoxification, metabolism, and protein synthesis. Hepatocytes have the ability to fuse with blood cells, generating hybrid hepatocytes that contain nuclei from both fusion partners. In cases of genetic liver disease, fusion between diseased hepatocytes and normal blood cells can result in the formation of hybrid hepatocytes that function normally. In this series of experiments, we show that fusion hepatocytes produce daughter cells with one-half the amount of DNA found in the parental fusion hepatocyte. Furthermore, we show that the daughter cells are genetically distinct from each other. The increase in genetic diversity within the liver could give rise to hepatocytes lacking proper growth control, potentially resulting in tumor formation and cancer.

## Introduction

Cell divisions in mitosis are thought to always produce daughter cells with the same chromosome content as the parental cell. Our recent studies with fusion-derived polyploid hepatocytes challenge that ideology. We propose that polyploid hepatocytes can undergo ploidy reductions, leading to the generation of genetically distinct daughter cells with reduced DNA content.

Our group previously showed that transplantation of wild-type bone marrow into *fumarylacetoacetate hydrolase* (*Fah*) knockout mice leads to the generation of fusion-derived hepatocytes [1],[2]. In this murine model for the human disease hereditary tyrosinemia type 1, hepatocytes expressing FAH have a strong selective growth advantage and can repopulate the diseased host liver [3],[4]. *Fah^−/−^* mice can be bred and kept healthy by administering the drug 2-(2-nitro-4-trifluoro-methylbenzol)-1,3-cyclohexanedione (NTBC) in their drinking water [5]. This drug blocks tyrosine catabolism upstream of FAH and, therefore, prevents the accumulation of fumarylacetoacetate, the toxic substrate of FAH. NTBC withdrawal induces liver injury and results in death from liver failure 4–8 weeks later. In bone marrow transplanted *Fah* ^−/−^ mice, fusion between the *Fah^+/+^* donor blood cells and *Fah^−/−^* host hepatocytes results in polyploid cells that have a selective advantage and can completely repopulate the liver [1],[2],[6]. Furthermore, FAH positive hepatocytes can be serially transplanted into secondary and tertiary *Fah* ^−/−^ recipients, thereby expanding the pool of hybrid hepatocytes and making them amenable to extensive genetic and cell biological analysis [3],[7]. Bone marrow transplantation has been shown to generate fusion-derived hepatocytes by numerous investigators [8]–[12]. However, direct differentiation of hematopoietic precursors into liver epithelial cells cannot be excluded, but it is clear that the majority of fusion-derived hepatocytes arise by fusion of donor blood cells with preexisting hepatocytes [1],[2],[6].

In our previous studies, chromosomal analysis of hepatocytes from bone marrow transplanted mice revealed the presence of diploid fusion-derived hepatocytes [1]. This result was surprising since fusion-derived cells should be at least tetraploid. Thus, we hypothesized that fusion-derived hepatocytes could undergo ploidy reductions during regeneration, leading to genetic diversity among daughter cells.

The present study rigorously examines whether fusion-derived hepatocytes undergo ploidy reductions. First, cell fusion experiments show conclusively that fusion-derived polyploid hepatocytes generate daughter cells with one-half DNA content. Unexpectedly, a high degree of aneuploidy was seen among fusion-derived cells. Secondly, ploidy reduction events were associated with independent marker segregation. Hepatocytes derived by cell fusion were expected to retain markers from each cell participating in the fusion event. Indeed, analysis of liver sections from repopulated mice showed the majority of nodules harboring both donor and host markers. However, we also detected a low (but highly reproducible) percentage of FAH positive nodules lacking additional markers. Third, to exclude the possibility that diploid hepatocytes expressing donor markers arose from transdifferentiation of hematopoietic cells, we employed a Cre-loxP reporter system in which ß-galactosidase (ß-gal) was only expressed in hepatocytes generated though cell fusion. As expected, polyploid hepatocytes expressed ß-gal and FAH. Consistent with the cytogenetic results, hepatocytes with diploid DNA content also expressed ß-gal and FAH. Finally, we tested individual cells for donor and host markers. Single cell PCR analysis of diploid daughter cells revealed a heterogeneous population containing a combination of donor and host markers. Taken together, our results demonstrate that fusion-derived polyploid hepatocytes undergo ploidy reduction events, generating heterogeneous populations of lower ploidy daughter cells.

## Results

### Fusion-Derived Hepatocytes Generate Daughter Cells with One-Half DNA Content

**Figure 1:**
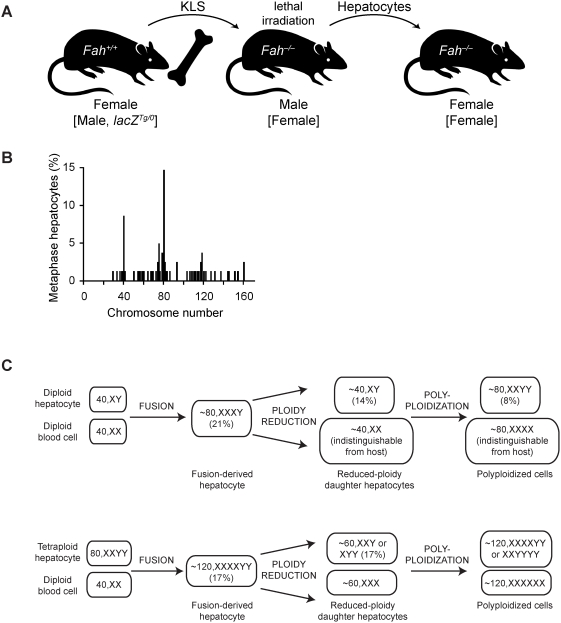
Hepatocyte-blood fusion generates fusion-derived hepatocytes and associated daughter cells. (A) Serial transplantation scheme for expansion of fusion-derived hepatocytes. KLS cells from female *Fah^+/+^* mice were transplanted into lethally irradiated *Fah^−^* ^/−^ male mice. Following liver repopulation, hepatocytes were isolated from primary hosts and serially transplanted into *Fah^−^* ^/−^ female mice (F>M>F). Alternatively, male KLS cells from ROSA26 mice (*lacZ^Tg/0^*) were transplanted into female recipients and subsequently serially transplanted into *Fah^−^* ^/−^ female mice (M>F>F). (B and C) Hepatocytes from serially transplanted mice (F>M>F) were karyotyped (n = 62 karyotypes from 4 transplanted mice). The distribution of Y-chromosome positive hepatocytes is shown (B). Fusion events between hepatocytes and blood cells gave rise to fusion-derived hepatocytes, which, in turn, underwent ploidy reductions (C). A fraction of reduced-ploidy daughter cells polyploidized. Parentheses indicate the percentage of metaphases scored with the indicated chromosome content.

Cell fusion produces hybrids with increased centrosome and chromosome numbers. Numerous studies have suggested that tetraploidy and aberrant centrosome numbers can result in genetic instability and cancer [13],[14]. To test whether hepatocytes generated by cell fusion *in vivo* are genetically stable, we karyotyped hepatocytes from serially transplanted mice. Lethally irradiated *Fah* ^−/−^ recipients were transplanted with cKit^+^ Lin^neg/lo^ Sca1^+^ (KLS) bone marrow cells from wild-type or ROSA26 (*lacZ^Tg/0^*) donors in a sex-mismatched fashion. Following NTBC withdrawal and liver repopulation, hepatocytes were serially transplanted into female *Fah* ^−/−^ recipients, allowing fusion-derived hepatocytes to undergo successive rounds of proliferation (Figure 1A). Importantly, only FAH positive cells (i.e., fusion products) but not *Fah^−/−^* hepatocytes can repopulate secondary recipient livers [3]. After completed repopulation, unselected hepatocyte metaphases were analyzed by standard G-banding techniques. Fusion between two diploid cells generates a tetraploid cell. Normal hepatocytes polyploidize in an age-dependent manner and can be diploid, tetraploid, octaploid or higher (reviewed in [13]). Therefore, the chromosome content of hybrid cells arising from hepatocyte-blood fusion must be tetraploid or greater.

The chromosome content of fusion-derived hepatocytes (i.e., positive for Y-chromosome) varied widely (Figure 1B). Approximately 15% of the metaphases had exactly 80 chromosomes with the percentage increasing to 30% of metaphases harboring nearly 80 chromosomes (80±5). Furthermore, 9% of metaphases had exactly 40 chromosomes, and the percentage increased to 14% containing nearly 40 chromosomes (40±4). The presence of diploid and nearly diploid fusion-derived hepatocytes suggests that they were produced by ploidy reduction events of tetraploid fusion products. Fusion hepatocytes with intermediate numbers of chromosomes were also found (>40 and <80, >80 and <160) (Figure 1B). Nearly all of the Y-chromosome containing metaphases had numerical chromosome abnormalities (Table 1). These numerical aberrations may have resulted from the fusion process or by DNA damage sustained by *Fah* ^−/−^ hepatocytes during NTBC withdrawal [15].

**Table 1:** Karyotypes of fusion-derived hepatocytes.

Transplant	Nearest Ploidy	Chromosome Number	Sex	Chromosome Gain	Chromosome Loss
Recipient #1	Diploid	40	XY	5, Mar1	17, 18
		40	XY	der(8), Mar1	18
	Triploid	55	XY	11, 15, 19	1, 4, 5, 6, 8, 10, 14, X or Y
		64	2X1Y	5×2, 9, 12×2, 15×2	6, 16, 19
		67	2X1Y	10, 11, 12, 14, 15, 17, 18, 19	8
	Tetraploid	79	3X1Y	None	6
		80	3X1Y	None	None
	Hexaploid	108	3X1Y	12, Mar1, Mar2×3	2, 3, 5×2, 6×2, 7, 8×2, 9, 10, 11, 14, 16×2, X, Y
		112	4X1Y	15×2, 16, Mar1, Mar2	2×2, 3×3, 6, 10×2, 11, 17, 19×2, Y
		117	4X1Y	8, 11, 12, 19×2, Mar1, Mar2, Mar3	3, 4, 7×2, 9×2, 10, 13, 14, 15, Y
Recipient #2	Diploid	38	XY	Mar1	1, 9, 14
		40	XY	2, Mar1×2	7, 9, 16
		40	XY	None	None
	Tetraploid	79	3X1Y	4, 18, Mar1×2	7, 8, 10, 12, 17
		80	2X2Y	1, 3, 18×3, 19×2	6, 7, 8×2, 9, 10×2
		80	3X1Y	3, 8	4, 9
		80	2X2Y	3×2, 4, 18, 19	5, 6×3, 10
		82	3X1Y	13, 17, 19×2, Mar1×3	1, 5, 16×3
	Hexaploid	117	5X1Y	11, 12, 19×2, X, Mar1×3	1, 2, 5, 6, 10, 13×2, 16, 17×2, Y
		122	4X2Y	1, 2, 19×2, Mar1	6, 7, 15

Representative karyotypes of fusion-derived hepatocytes (Y-chromosome positive) from two independent serial transplant recipients (F>M>F). Chromosome gains and losses are described relative to the nearest cell ploidy level.

The distribution of chromosomes among fusion-derived hepatocytes clearly supported a pattern of cell fusion, ploidy reduction and polyploidization (Figure 1C). For example, fusion between a female diploid blood cell (40,XX) and male diploid hepatocyte (40,XY) results in a tetraploid fusion-derived hepatocyte (80,XXXY). A ploidy reduction event generates two types of daughter cells (40,XY and 40,XX) that can polyploidize, giving rise to tetraploid cells (80,XXYY and 80,XXXX, respectively). Because we could not distinguish between daughter cells that lost the Y-chromosome and host female hepatocytes, we focused exclusively on Y-chromosome positive metaphases. Fusion-derived tetraploid hepatocytes (∼80,XXXY) were detected in 21% of the metaphases analyzed. Approximately 14% of Y-chromosome positive metaphases were nearly diploid (∼40,XY), corresponding to daughter cells arising through ploidy reduction. Furthermore, 8% of cells were ∼80,XXYY, which is the expected karyotype for polyploidized diploid cells.

In addition to finding fusion-derived hepatocytes arising from diploid-diploid cell fusion, we detected cells generated by fusion between male tetraploid hepatocytes (80,XXYY) and female diploid blood cells (40,XX). Hexaploid cells (120,XXXXYY) were detected in 17% of the metaphases. Furthermore, triploid cells (either ∼60,XYY or XXY), which are the predicted daughter cells of hexaploid reduction events, were identified in 17% of metaphases. Together, these data strongly support the emergence of daughter cells containing one-half DNA content from fusion-derived hepatocytes. Similar to normal diploid hepatocytes, diploid daughter cells either remain diploid or polyploidize to generate tetraploid hepatocytes.

### Marker Segregation in Fusion-Derived Hepatocytes

**Figure 2:**
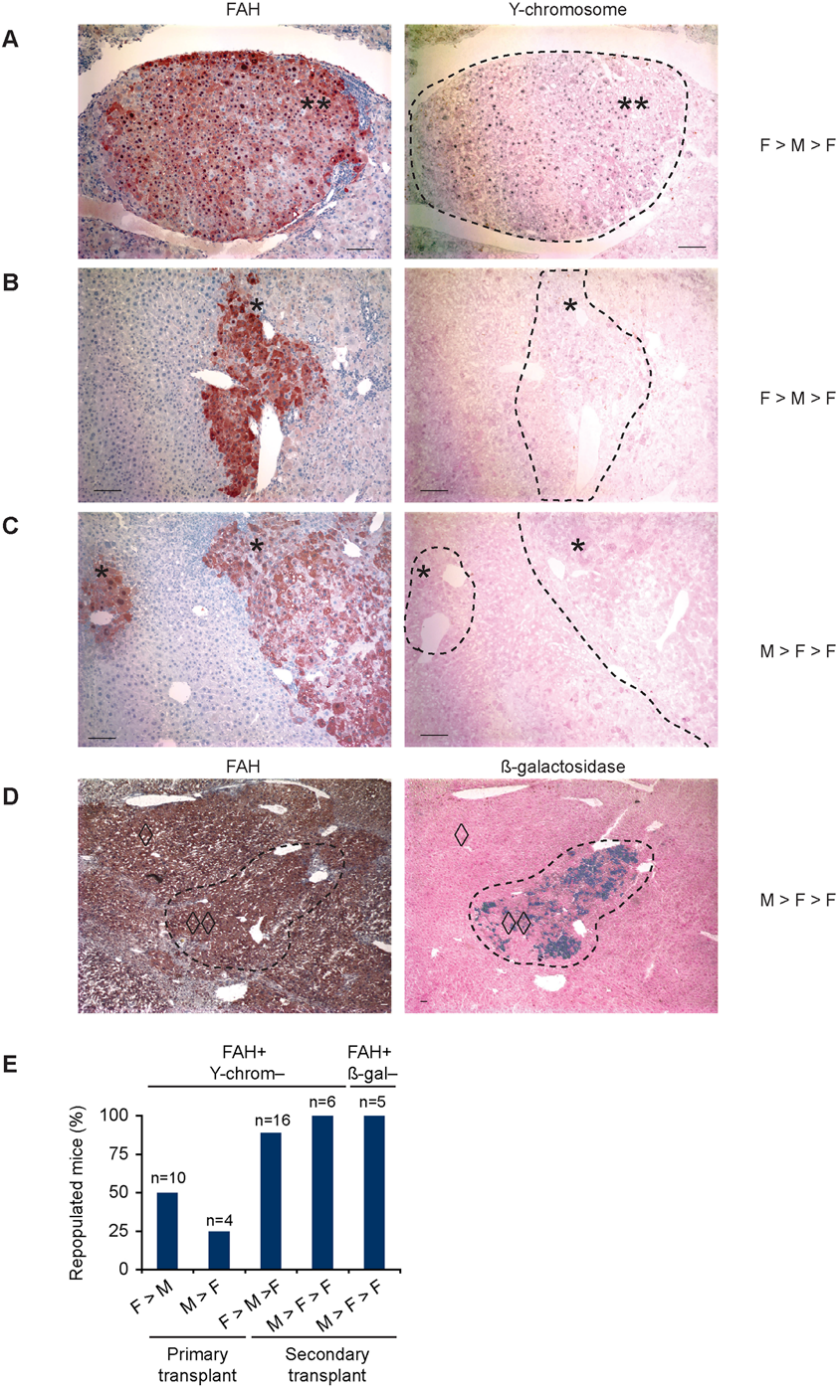
Marker loss in livers repopulated with fusion-derived hepatocytes. (A–D) Marker analysis was performed on sequential liver sections from serially transplanted mice. The transplantation scheme (F>M>F or M>F>F) is indicated. FAH+ nodules (brown) either co-expressed the Y-chromosome (black dots) (A) or were devoid of Y-chromosome staining (B and C). Double asterisks (**) indicate double positive nodules (FAH+ and Y-chromosome+) whereas single asterisks (*) indicate single positive nodules (FAH+ and Y-chromosome−). (D) ß-gal (blue staining) is expressed in a single FAH+ nodule but is absent from peripheral FAH+ tissue. Double diamonds (◊◊) indicate double positive tissue (FAH+ and ß-gal+) and single diamonds (◊) indicate a single positive nodule (FAH+ and ß-gal−). Scale bars are 200 µm. (E) Percentage of transplanted mice containing FAH+ fusion-derived nodules that lack Y-chromosome or ß-gal is shown.

After demonstrating that fusion-derived hepatocytes could generate daughter cells with one-half chromosome content, we hypothesized that ploidy reduction events should also lead to marker segregation among daughter cells. If ploidy reduction occurs in a tetraploid fusion-derived hepatocyte, there is a 50% chance of losing a heterozygous or hemizygous marker. Liver sections from mice repopulated by fusion-derived hepatocytes in serial transplantation experiments (Figure 1A) were stained for FAH, Y-chromosome and ß-gal activity (Figures 2A–2D). Typically, FAH is co-expressed with the Y-chromosome (Figure 2A) and ß-gal (Figure 2D). However, while most regenerating nodules expressed all markers of cell fusion, a fraction of FAH positive nodules (2–5%) were Y-chromosome negative (Figures 2B and 2C) or ß-gal negative (Figure 2D). Based on the expected loss-of-heterozygosity frequency of one-half, the observed frequency suggests that 4–10% of the FAH positive nodules were initiated by cells that had undergone ploidy reductions.

Heterogeneous FAH positive nodules (lacking one or more donor markers) were consistently found in repopulated mice (Figure 2E). Similar results were obtained regardless of the transplantation scheme (F>M>F or M>F>F). Approximately 25–50% of primary recipients contained Y-chromosome negative FAH positive nodules, and this number increased to 90–100% of secondary recipients. ß-gal negative FAH positive nodules were seen in all serially transplanted mice analyzed. These data demonstrate that ploidy reduction events leading to the formation of heterogeneous daughter cells occur in a large fraction of livers repopulated by fusion-derived hepatocytes.

### Ploidy Reduction in Fusion-Derived Hepatocytes

**Figure 3:**
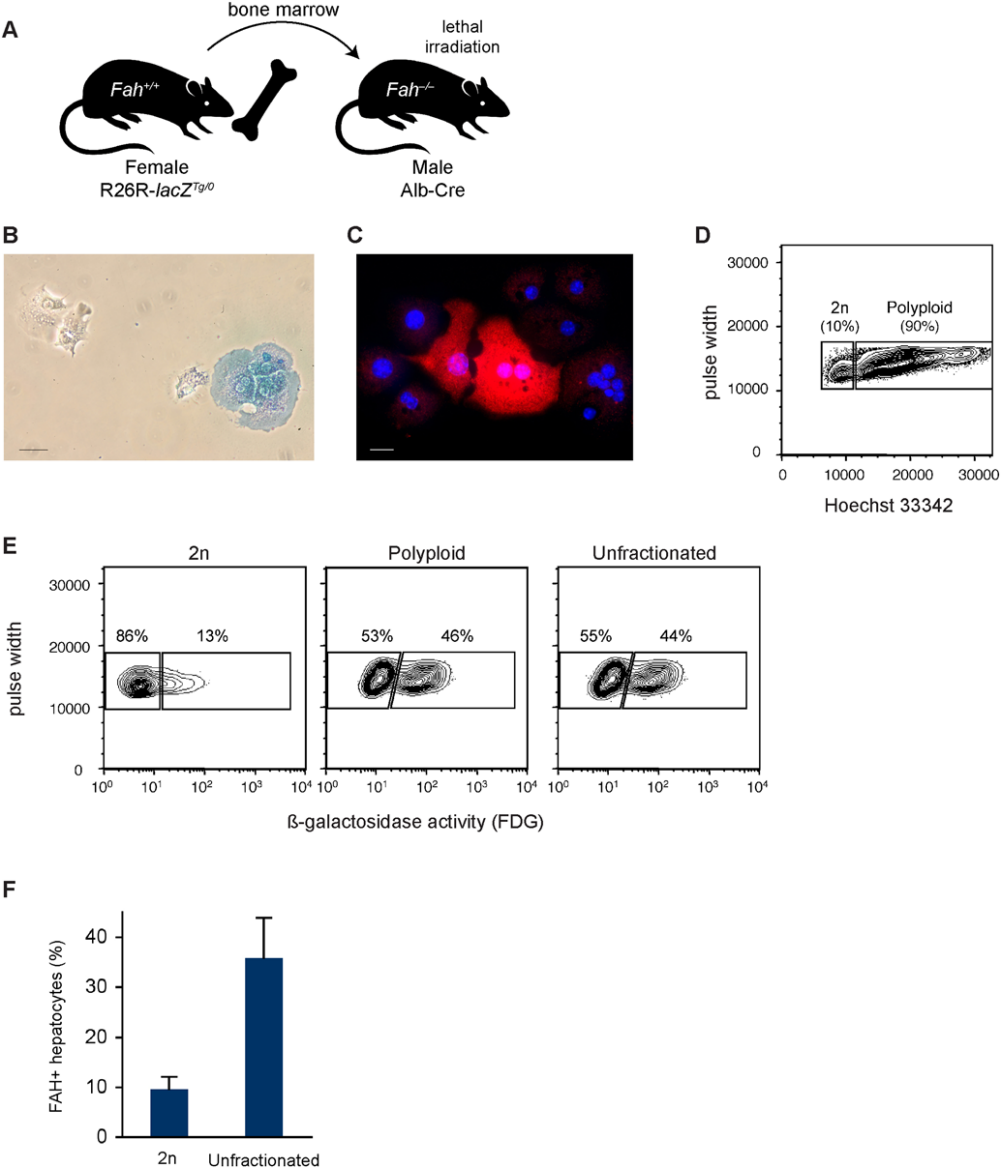
Diploid hepatocytes express markers of cell fusion. (A) Transplantation scheme for detection of fusion-derived hepatocytes. Donor bone marrow cells (from female transgenic mice hemizygous for R26R) were transplanted into lethally irradiated male *Fah^−/−^* mice containing the Alb-Cre transgene. Following fusion between donor hematopoietic cells and host hepatocytes, loxP sites were excised by Cre recombinase, leading to ß-gal expression via the ROSA26 promoter. (B and C) Hepatocytes from repopulated mice expressed ß-gal (blue, as seen by X-Gal staining) (B) and FAH (red) (C). Scale bars are 100 µm (n = 5). (D and E) Hepatocyte suspensions were loaded with Hoechst 33342 and FDG. Livers from repopulated mice were comprised of diploid and polyploid hepatocytes (D). FACS analysis shows the ploidy distribution from a representative mouse (n = 8). ß-gal was expressed by a subset of each hepatocyte ploidy population (E). FACS analysis shows FDG expression by diploid, polyploid and unfractionated hepatocytes from a representative liver (n = 5). (F) FAH was expressed by unfractionated hepatocytes as well as FACS-sorted diploid hepatocytes. Data represent percentage (average±SEM) of cells expressing FAH (n = 4).

To facilitate the genetic analysis of fusion-derived hepatocytes, a Cre-loxP system was used to track fusion products. *Fah* ^−/−^ mice were bred with transgenic animals expressing Cre-recombinase via a hepatocyte-specific albumin promoter (Alb-Cre) [16]. Lethally-irradiated recipient mice were transplanted with bone-marrow from a ROSA26 reporter (R26R) mouse (Figure 3A) [17]. These animals harbor a floxed allele of the *lacZ* gene at the ROSA26 locus. In this transplantation scheme, ß-gal is only expressed when the R26R and Alb-Cre alleles are combined in the same cell. Hepatocytes were isolated from repopulated mice and found to express ß-gal, conclusively showing that these cells were derived by fusion between donor blood cells and host hepatocytes (Figure 3B). Fusion-derived cells also expressed FAH, indicating the successful activation of wild-type *Fah* supplied by donor hematopoietic cells (Figure 3C).

Next, we examined reduction of DNA content and marker segregation in fusion hepatocytes. Single cell hepatocyte suspensions (containing a mixture of fusion-derived hepatocytes and host hepatocytes) from repopulated mice were loaded with the DNA dye Hoechst 33342 and analyzed by flow cytometry. In control experiments, hepatocyte ploidy populations were readily identified in non-transplanted mice (Figure S1A and S1B). As expected, livers isolated from aged mice (Figure S1A) contained fewer diploid hepatocytes than livers isolated from young mice (Figure S1B). Furthermore, diploid hepatocytes were isolated with high purity. Sorted 2n hepatocytes were >99% pure (Figure S1C), and they contained a single Y-chromosome (Figure S1D). Analysis of mice repopulated to ∼40% by fusion hepatocytes revealed populations of diploid and polyploid hepatocytes (Figure 3D). The diffuse polyploid population is consistent with the chromosome distribution in Figure 1B. To utilize ß-gal as a marker of cell fusion, hepatocytes were loaded with fluorescein di-ß-D-galactopyranoside (FDG), a substrate that becomes fluorescent when cleaved by the enzyme. As expected from the overall degree of FAH repopulation, ß-gal was expressed by a fraction of polyploid hepatocytes (27±11% of all cells) (Figure 3E). Moreover, interrogation of diploid hepatocytes revealed a subpopulation of ß-gal positive cells (8±3% of all diploids). Hepatocyte populations of different ploidy were also FACS-sorted and subjected to FAH immunocytochemistry (Figure 3F). A portion of unfractionated hepatocytes expressed FAH (36±8%). Consistent with ß-gal expression, a subpopulation of diploid hepatocytes also expressed FAH (10±3%). Together, these results show that fusion-derived hepatocytes undergo ploidy reduction events, generating diploid hepatocytes that express ß-gal and FAH.

### Marker Loss in Reduced-Ploidy Daughter Cells

Although the mechanism by which fusion-derived hepatocytes undergo ploidy reductions is unknown, the data clearly suggest a model in which individual chromosomes/markers segregate independently of one another. For example, as described in Figure 3A, fusion between a donor diploid blood cell (female, R26R^Tg/0^, *Fah^+/+^*) and recipient diploid hepatocyte (male, Alb-Cre, *Fah^−/−^*) generates a tetraploid cell containing a single Y-chromosome and a single copy of R26R. The Alb-Cre genotyping assay fails to distinguish between hemi- and homozygous mice. Thus, tetraploid fusion-derived hepatocytes undergoing ploidy reductions should generate a pair of diploid daughter cells, and each daughter cell should have a 50% chance of inheriting either R26R or the Y-chromosome.

**Figure 4:**
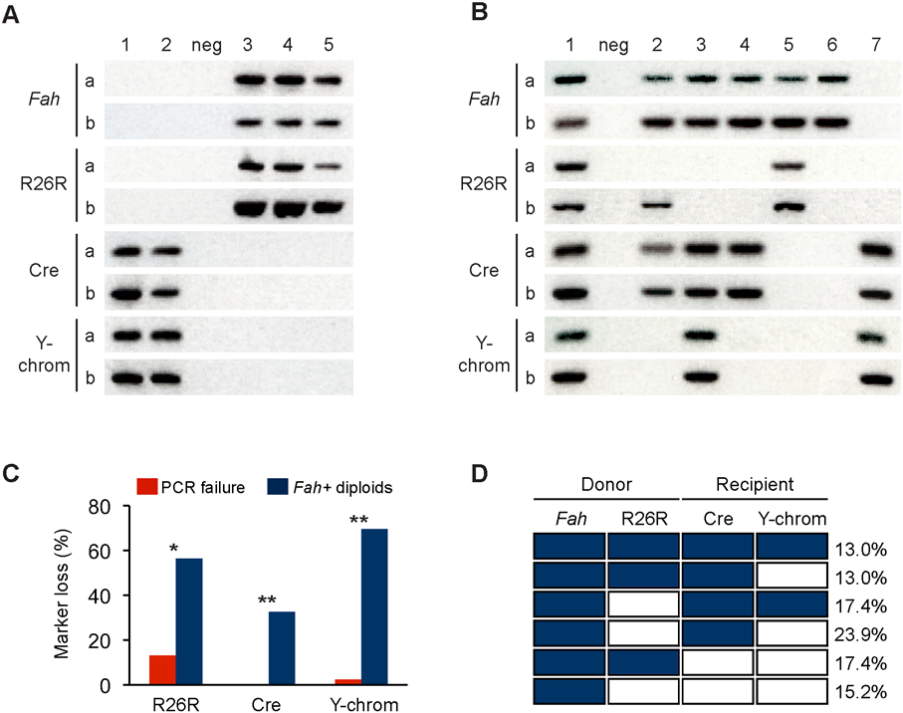
Fusion markers segregate independently in diploid daughter hepatocytes. FACS-isolated cell populations from mice repopulated via the scheme in Figure 3A were genotyped by single cell PCR for donor (*Fah* and R26R) and host (Cre and Y-chromosome) markers. Two primer sets per marker were utilized (i.e., primer sets “a” and “b”). (A) Representative data for splenocytes is shown (n = 100 cells from 4 repopulated mice). Cells 1 and 2 were positive for host markers and cells 3–5 were positive for donor markers. Negative control is marked “neg.” (B) Representative data for diploid hepatocytes is shown (n = 157 cells from 2 repopulated mice). Cell 1 was positive for all markers, whereas cell 7 was positive for only recipient markers. Cells 2 and 3 were negative for the Y-chromosome and Cre transgene, respectively. An example of PCR failure is shown for cell 2 (R26R). Although this cell was negative for R26R-a, it was positive for R26R-b. Therefore, cell 2 was scored “positive” for R26R. Loss of two or more markers was seen in cell 4 (R26R/Y-chromosome), cell 5 (Cre/Y-chromosome) and cell 6 (R26R/Cre/Y-chromosome). (C and D) *Fah+* diploid hepatocytes were genotyped (n = 44 cells from two mice). The percentage of single cells that lost R26R, Cre and the Y-chromosome is compared to the calculated PCR failure rate for each marker (C). *, *P* = 0.006; ** *P*<0.0001. The presence (shaded boxes) and absence (white boxes) of donor and host markers is shown for each subpopulation (D). The percentage of each subpopulation is indicated.

We performed single cell genotyping of diploid hepatocytes to determine whether chromosomes/markers were lost in cells that had undergone ploidy reduction events. Hepatocytes from repopulated livers (Figure 3A) were FACS-purified on the basis of DNA content and genotyped for donor (*Fah* and R26R) and host (Cre and Y-chromosome) markers. One of the major obstacles to single cell genotyping is PCR failure, resulting from DNA degradation or template inaccessibility [18]. In our hands, PCR failure ranged from 0 to 40%, which is consistent with published rates [18]. To minimize the effects of PCR failure, each marker was detected with two independent primer sets, thus reducing the net dropout rate to 0% (*Fah*), 13.2% (R26R), 0.5% (Cre) and 2.5% (Y-chromosome). As a control, single splenocytes from repopulated mice were genotyped and found to contain only host markers (cells 1 and 2) or donor markers (cells 3–5) (Figure 4A). These results are consistent with a high degree of donor engraftment seen in our KLS and bone marrow transplanted mice (Duncan, Hickey and Grompe, unpublished results).

Single cell genotyping was performed on 157 diploid hepatocytes derived from two independently transplanted mice. Representative PCR data is shown (Figure 4B). Cell 7 contained only host markers, suggesting that it was host-derived. Cell 1, which was positive for all makers, was fusion-derived. All of the remaining cells illustrated loss of one (cells 2 and 3) or more markers (cells 4–6). Overall, *Fah* was detected in 29% of the cells. Detailed analysis of the diploid *Fah* positive fusion products revealed that 57% lost the R26R transgene, 33% lost the Cre transgene and 70% lost the Y-chromosome (Figure 4C). Although PCR failure may account for a small percentage of the observed marker loss, the high degree of marker loss represents loss-of-heterozygosity at the indicated locus. Furthermore, loss of one or more markers was identified in subpopulations of diploid *Fah* positive hepatocytes (Figure 4D). Only 13% of cells, for example, contained all four markers. Loss of a single marker was detected in 13% (Y-chromosome) and 17% (R26R) of the cells. Loss of two markers (R26R/Y-chromosome, Cre/Y-chromosome) or three markers (R26R/Cre/Y-chromosome) was found in 24%, 17% and 15%, respectively, of diploid hepatocytes. Together, these data showed that diploid daughter cells were genetically unique, suggesting that autonomous markers segregate independently during ploidy reduction events.

## Discussion

In this study, we demonstrated that fusion-derived hepatocytes could undergo ploidy reductions. Initially, serial transplantation experiments were performed. Cytogenetic analysis showed that 14% of fusion-derived hepatocytes were nearly diploid. Surprisingly, fusion-derived hepatocytes were highly aneuploid. While most regenerating nodules expressed all markers of cell fusion, a fraction of FAH positive nodules (2–5%) were Y-chromosome negative or ß-gal negative. This frequency suggests that 4–10% of the nodules were initiated by cells that had undergone ploidy reduction events. Next, we utilized a ß-gal reporter system to track fusion products in discrete ploidy populations. Polyploid hepatocytes expressed FAH (indicating that fusion-derived cells were reprogrammed to express donor genes) and ß-gal (demonstrating that these cells were derived by cell fusion). Significantly, diploid hepatocytes also expressed ß-gal and FAH, establishing that these cells originated from polyploid fusion-derived hepatocytes. Finally, we carefully tracked donor/host markers in hepatocytes that had undergone ploidy reductions by single cell genotyping. The majority of diploid daughter hepatocytes (87%) were negative for one or more markers, giving rise to a heterogeneous population of cells. These results suggest that markers/chromosomes segregate independently during ploidy reduction events.

Hepatocyte polyploidization has been documented in many species (reviewed by Gupta [13]), but ploidy reversal has not been rigorously characterized. Our experiments provide proof-of-concept that ploidy reversal does occur in fusion-derived hepatocytes. Several reports also suggest that normal hepatocytes may undergo ploidy reduction. For instance, treatment of rodents with hepatotoxins thioacetamide [19] and carbon tetrachloride [20] led to a dramatic increase in diploid hepatocytes and concomitant decrease in polyploid hepatocytes over 72 hr. Differential proliferation and/or cell death was not seen among diploid or polyploid hepatocytes [20]. Thus, it is possible that normal polyploid hepatocytes undergo ploidy reductions, but this hypothesis remains to be tested.

The high degree of aneuploidy displayed by fusion-derived hepatocytes is surprising. It is unclear whether aneuploidy resulted directly from the fusion and/or ploidy reduction events or indirectly as a consequence of the *Fah* repopulation model [5]. Furthermore, we cannot exclude the possibility of stochastic chromosome loss during mitosis [21]. Thus, aneuploid hepatocytes could arise from the random loss of chromosomes by fusion-derived hepatocytes undergoing extensive proliferation.

**Figure 5:**
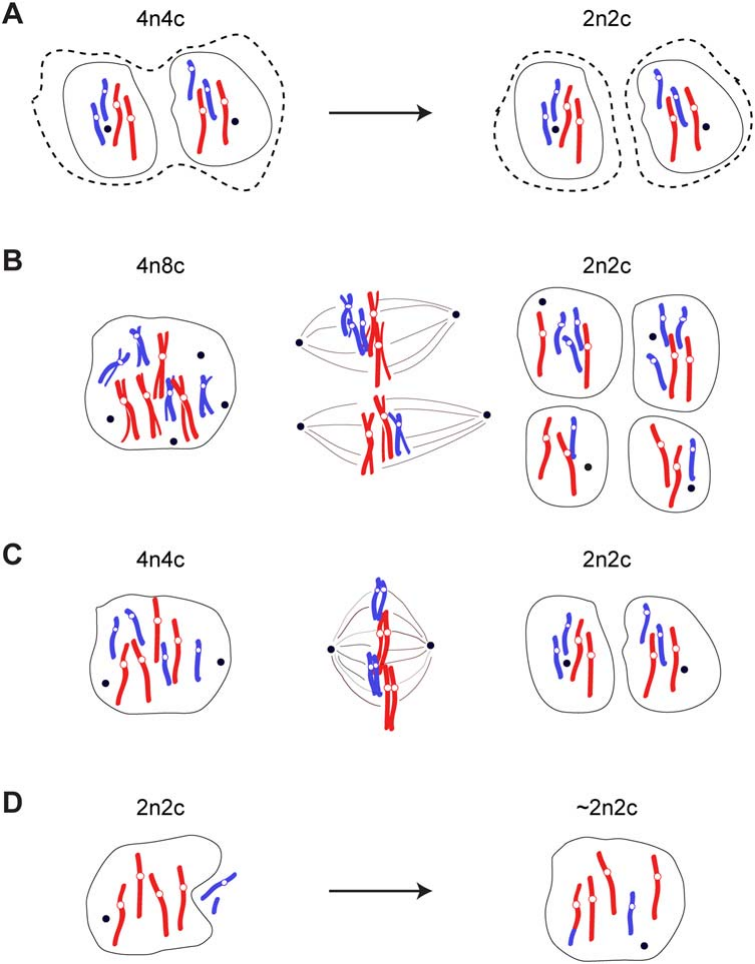
Potential mechanisms for diploid hepatocyte formation from polyploid fusion-derived hepatocytes. (A) Cytokinesis without mitosis. A binucleated cell undergoes cytokinesis before entering the next cell cycle. This process would not produce marker loss or aneuploidy. (B) Multiple spindles, followed by multipolar (in this case tetrapolar) mitosis. Extreme aneuploidy would result. (C) Mitosis without S-phase with chromosome pairing. This would ensure proper chromosome segregation and would facilitate distribution of hemizygous markers between daughter cells. (D) Horizontal gene transfer. A diploid cell engulfs a neighboring cell undergoing apoptosis. Single chromosomes and/or chromosome fragments would be incorporated into the nucleus while maintaining a nearly diploid karyotype. The parental cell is shown at left, mitotic spindle(s) (when necessary) in the middle and resulting diploid cells on the right. Open circles represent centromeres. Black circles represent centrosomes. Chromosomes are shown in different colors to indicate their lineage. Host hepatocyte chromosomes are red, and donor chromosomes are blue.

A number of possibilities could explain how diploid hepatocytes are generated from polyploid fusion-derived hepatocytes. First, it is theoretically possible that binucleated fusion-derived hepatocytes could simply complete cytokinesis (Figure 5A). Normal binucleated polyploid hepatocytes are formed through failed cytokinesis [22],[23]. For example, a mononucleated diploid hepatocyte undergoes a regular mitosis, but then separation of the two daughter cells fails, generating a binucleated tetraploid cell with two diploid nuclei [22]. Whether binucleated hepatocytes could resume cytokinesis is unclear, but it remains a possibility. In the context of fusion-derived hepatocytes, the completion of cytokinesis would generate two mononucleated diploid daughter cells, each with the same genotype as the original fusion partners. As seen in Figure 4D, subsets of diploid hepatocytes contained a donor marker (*Fah*) and a recipient marker (Cre and/or Y-chromosome), proving that these cells were genetically distinct from the original fusion partners. Therefore, a cytokinesis-type mechanism can be excluded.

The second possibility is chromosome loss via multipolar mitosis, which can lead to the random segregation of chromosome content among two or more daughter cells [24]. Fusion-derived hepatocytes have increased numbers of centrosomes, which could result in the formation of multiple spindle poles during prophase. Thus, multipolar mitotic events could enrich for daughter cells with diploid chromosome content (Figure 5B). However, multipolar mitosis cannot adequately explain the clustering of fusion-derived hepatocytes with atypical chromosome counts. For example, triploid hepatocytes with ∼60 chromosomes (XXY or XYY) comprised 17% of the metaphases analyzed (Figure 1C). These daughter cells likely originated from hexaploid fusion-derived hepatocytes. It is difficult to imagine how multipolar mitosis would enrich for cells with such abnormal chromosome counts. Furthermore, if ploidy reduction were achieved by multipolar mitosis, then each chromosome should be lost with the same low frequency (i.e., 1/19 for autosomes). Single cell genotyping of diploid daughter hepatocytes showed loss of R26R (located on chromosome 6 [25]) and the Y-chromosome at 50% or greater (Figure 4C). This high degree of marker segregation strongly suggests that chromosome/marker loss occurs in a non-random fashion.

Another possibility to explain the emergence of daughter hepatocytes with one-half DNA content is cell division without DNA replication (Figure 5C). This type of ploidy reduction was first described in the mosquito *Culex pipiens* [26],[27] but has never been described in mammalian cells. In this model, fusion-derived hepatocytes could proceed through G1 phase of the cell cycle, skip S-phase and progress to G2/mitosis. Pairing between homologous chromosomes would ensure proper chromosome segregation. This type of mechanism accounts for the generation of diploid daughter cells (Figures 1B, 3E and 3F) as well as enrichment for atypical triploid daughter cells (Figure 1B). Moreover, the high degree of marker loss seen in diploid daughter cells (Figure 4C) is possible through a chromosome pairing interaction. Rigorous testing of all potential mechanisms must be performed to elucidate the cellular processes governing ploidy reductions in fusion-derived hepatocytes.

Finally, direct transmission of DNA via horizontal gene transfer (HGT) into diploid hepatocytes must be considered. HGT among somatic cells involves phagocytosis of apoptotic cells followed by nuclear uptake/integration of whole chromosomes or chromosome fragments by the engulfing cell [28],[29]. HGT was hypothesized to induce hepatocyte reprogramming in xenotransplantation experiments [30]. In our studies, diploid host hepatocytes could acquire genes from apoptotic donor blood cells, resulting in hepatocyte reprogramming while maintaining a nearly diploid chromosome count (Figure 5D). However, the presence of multiple donor markers on different chromosomes by HGT is expected to be rare, and we found that nearly half of diploid fusion-derived hepatocytes harbored at least two donor markers (Figure 4D). Therefore, our data strongly argue against an HGT-type of mechanism.

Regardless of the mechanism, ploidy reduction events have significant implications. In the context of fusion-derived hepatocytes, ploidy reductions can be a confounding factor when tracing markers during stem cell transplantation. Donor markers can be lost during ploidy reductions, thus leading to an underestimate of engraftment. Similarly, host markers can be lost from hybrids, obscuring the existence of fusion and giving the false impression of transdifferentiation. Because cell fusion between transplanted cell types and target organs has been described in many experimental systems (reviewed in [31]), the possibility of ploidy reductions needs to be considered when interpreting cell transplantation experiments. Furthermore, we propose that ploidy reduction events may contribute to tumorigenesis. The independent segregation of chromosomes from polyploid cells results in genetically heterogeneous diploid daughter hepatocytes. Individual daughter cells could lose tumor suppressors, generating a subset of hepatocytes with oncogenic potential.

## Methods

### Ethics Statement

The Institutional Animal Care and Use Committee of the Oregon Health and Science University approved all mouse experiments.

### Mice

The following inbred mouse strains were used: wild-type (C57Bl and 129), transgenic ROSA26 (C57Bl and 129) [32], *Fah^−/−^* (C57Bl and 129) [33], transgenic R26R-*lacZ* C57Bl [34], transgenic Albumin-Cre C57Bl [35].

### Cell Isolation and Sorting

KLS cells were sorted from mouse bone marrow, as described [36]. Antibodies used for cell sorting are described in Protocol S1.

Primary hepatocytes were isolated by two-step collagenase perfusion [4] and cultured hepatocytes were isolated by trypsinization. For detection of hepatocyte ploidy populations, hepatocytes (2×10^6^/ml) were incubated with 15 µg/ml Hoechst 33342 (Sigma) and 5 µM reserpine (Invitrogen) for 30 min at 37°. Cells were analyzed and/or sorted with an InFlux flow cytometer (Cytopeia) using a 150 µm nozzle. Dead cells were excluded on the basis of 5 µg/ml propidium iodide (Invitrogen) incorporation. Cells adhering to each other (i.e., doublets) were eliminated on the basis on pulse width. Ploidy populations were identified by DNA content using an ultraviolet 355 nm laser and 425–40 nm bandpass filter. Sorted hepatocytes were collected in DMEM with 4.5 g/l glucose (HyClone) containing 50% fetal bovine serum (FBS) (HyClone). The purity of sorted populations was determined at the end of each sort, and only highly purified populations (>99% pure) were used for subsequent assays.

### Transplantation

Transplantation of hematopoietic cells (either bone marrow or KLS cells) was performed as previously described [1],[12]. Briefly, hematopoietic cells (either 4×10^6^ bone marrow cells or 3–4×10^3^ KLS cells) were injected retro-orbitally into groups of congenic *Fah* ^−/−^ recipient mice. KLS cells were co-transplanted along with 3×10^5^ bone marrow cells derived from an unirradiated recipient mouse. Host mice were lethally irradiated with 12 Gy using a ^137^Cs irradiator 4–24 hr prior to transplantation, obtained by two doses of 6 Gy each. Mice were maintained on NTBC drinking water (8 µg/ml). NTBC was withdrawn 3–12 weeks post transplantation, providing a selective environment for the proliferation of FAH positive fusion-derived hepatocytes.

For serial transplantation experiments, hepatocytes were isolated from primary transplanted mice and 1–3×10^5^ cells injected intrasplenically into *Fah^−/−^* recipient mice. NTBC was stopped immediately, allowing for selection of FAH positive cells [3].

### Cytogenetics

Freshly isolated primary hepatocytes were seeded at 1–2×10^3^ cells/cm^2^ on Primaria tissue culture plastic (Beckton Dickinson). Cells were incubated in hepatocyte culture medium containing DMEM with 4.5 g/l glucose (HyClone), 10% FBS (HyClone), nonessential amino acids (Cellgro) and antibiotic-antimycotic (Cellgro). Nonadherent cells were removed after 4 hr. Hepatocytes were then incubated in culture medium supplemented with 100 ng/ml human epidermal growth factor (Invitrogen) plus insulin, transferrin, selenium and ethanolamine (ITS-X, Invitrogen). After ∼40 hr, hepatocytes were treated with 150 mg/ml colcemid (Sigma) for 2–4 hr and harvested by trypsinization. After extensive washing, slides were incubated for 10 min in 56 mM KCl with 5% FBS and fixed with methanol:acetic acid (3∶1 ratio). For karyotype analysis, chromosomes were G-banded with a standard trypsin/Wright's stain protocol. Fluorescent in situ hybridization on interphase hepatocytes was performed using a Cy3-labeled whole chromosome paint (mouse Y-chromosome) per manufacturer's instructions (Cambio).

### Histology and Immunocytochemistry

Histological analyses were performed as described [3]. For FAH immunocytochemistry, hepatocytes (either unfractionated or FACS-purified) were allowed to adhere to collagen-coated Lab-Tek II, CC2-treated chamber slides (Nunc) in hepatocyte culture medium for 24 hr. Slides were washed extensively, fixed with methanol and dehydrated with acetone. After blocking in 5% normal donkey serum, cells were incubated with a custom rabbit polyclonal FAH antibody diluted 1∶1000 and detected with 10 ng/ml donkey anti-rabbit secondary antibody conjugated to Alexafluor 555 (Invitrogen). Nuclei were visualized with 200 ng/ml Hoechst 33342. For detection of ß-gal activity, hepatocytes were plated in culture medium on Primaria plastic. After 24 hr, adherent hepatocytes were subjected to X-Gal (5-bromo-4-chloro-3-indolyl-ß-D-galactopyranoside, Invitrogen) staining as described [37]. Alternatively, flow cytometry was used to detect ß-gal activity in Hoechst-stained hepatocytes (for the detection of ploidy populations) using FDG reagent (Invitrogen) per manufacturer's instructions.

### Single-Cell PCR

Single hepatocytes or splenocytes were FACS-sorted into individual wells of a 96-well PCR plate, lysed and subjected to semi-nested PCR, as described in Protocol S2.

### Statistical Analysis

Fisher's exact test (two-sided) was used to determine statistical significance. *P* values less than 0.05 were considered statistically significant.

## Supporting Information

Figure S1 Hepatocytes are FACS-isolated with high purity. (A and B) Hepatocytes isolated from 4 month old (A) and 20 day old (B) male non-transplanted mice were loaded with Hoechst 33342 and analyzed by flow cytometry. FACS plots show representative ploidy distributions (n>10 for 3–5 month old mice; n = 3 for 20 day old mice). (C and D) Purity of FACS-isolated diploid hepatocytes (from the 20 day old mouse) was evaluated. Sorted diploid hepatocytes were >99% pure, as detected by FACS (n>10) (C). All sorted diploid hepatocytes contained a single Y-chromosome (red), which is expected for diploid male cells (D). Nuclei are shown in blue. Scale bar is 20 µm (n = 5).
(0.34 MB TIF)

Protocol S1 KLS cell sorting from mouse bone marrow.
(0.03 MB PDF)

Protocol S2 Single cell PCR.
(0.09 MB PDF)
