# Supplementary figures and images for: Ploidy Reductions in Murine Fusion-Derived Hepatocytes
Source: PLoS Genet. 2009 Feb 20;5(2):e1000385. doi: 10.1371/journal.pgen.1000385 (PMC2636893; doi:10.1371/journal.pgen.1000385)

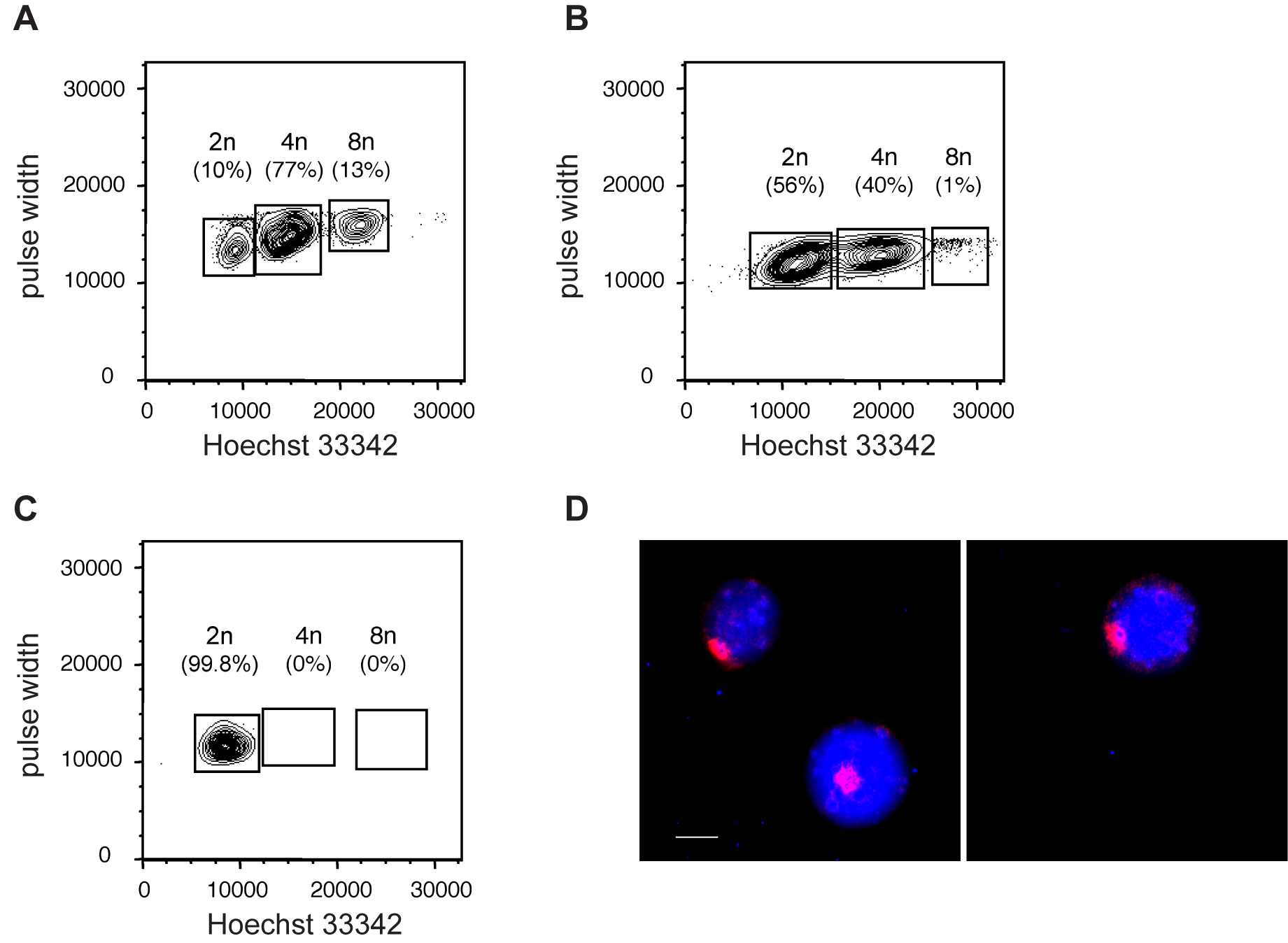

Supplement: Figure S1 — Hepatocytes are FACS-isolated with high purity. (A and B) Hepatocytes isolated from 4 month old (A) and 20 day old (B) male non-transplanted mice were loaded with Hoechst 33342 and analyzed by flow cytometry. FACS plots show representative ploidy distributions (n>10 for 3–5 month old mice; n = 3 for 20 day old mice). (C and D) Purity of FACS-isolated diploid hepatocytes (from the 20 day old mouse) was evaluated. Sorted diploid hepatocytes were >99% pure, as detected by FACS (n>10) (C). All sorted diploid hepatocytes contained a single Y-chromosome (red), which is expected for diploid male cells (D). Nuclei are shown in blue. Scale bar is 20 µm (n = 5). (0.34 MB TIF) [file pgen.1000385.s001.tif]
