# Supplementary material for: Ploidy Reductions in Murine Fusion-Derived Hepatocytes
Source: PLoS Genet. 2009 Feb 20;5(2):e1000385. doi: 10.1371/journal.pgen.1000385 (PMC2636893; doi:10.1371/journal.pgen.1000385)
Supplement: Protocol S1 — KLS cell sorting from mouse bone marrow. (0.03 MB PDF) [file pgen.1000385.s002.pdf]

Duncan et al.
Ploidy Reductions in Murine Fusion-derived Hepatocytes
PLoS Genetics, 2009

Protocol S1: KLS cell sorting from mouse bone marrow

KLS cells were sorted and re-analyzed based on surface marker expression of c-Kit and Sca-1 and low to negative expression of lineage markers. The combination of the following antibodies defined the lineage markers: 145-2C11 (anti-CD3e), 53-7.3 (anti-CD5), GK1.5 (anti-CD4), 53-6.7 (anti-CD8), RB6-8C5 Ly-6G (anti-Gr-1), M1/70 (anti-CD11b, Mac-1), Ter119 (anti-erythrocyte specific antigen), PK136 (anti-NK1.1) and 6B2 (anti-B220). Other antibodies used included clones 2B8 (anti-CD117, c-Kit) and D7 (anti-Ly-6A /E, Sca-1). All antibodies were purchased from Pharmingen or eBioscience. Analysis and cell sorting were carried out with an InFlux flow cytometer (Cytopeia) using a 70 μm nozzle.
