# Supplementary material for: Ploidy Reductions in Murine Fusion-Derived Hepatocytes
Source: PLoS Genet. 2009 Feb 20;5(2):e1000385. doi: 10.1371/journal.pgen.1000385 (PMC2636893; doi:10.1371/journal.pgen.1000385)
Supplement: Protocol S2 — Single cell PCR. (0.09 MB PDF) [file pgen.1000385.s003.pdf]

Duncan et al.
Ploidy Reductions in Murine Fusion-derived Hepatocytes
PLoS Genetics, 2009

Protocol S2: Single cell PCR

Single hepatocytes or splenocytes were FACS-sorted into individual wells of a 96-well PCR plate containing 5 µl lysis solution (4.7 µl 0.005% SDS, 0.2 µl 10mg/ml proteinase K, 0.1 µl 0.5M EDTA). Following incubation at 50°C for 30 min and subsequent denaturation of proteinase K at 99°C for 5 min, a semi-nested PCR was performed. Initially, multiplex PCR was carried out with primers for all loci. First round PCR was performed in a final volume of 50 µl containing 0.4 µM of each primer (external forward primers and common reverse primers), 0.2 mM dNTPS, 1 mM $MgCl_2$ and 0.75U *Taq* Polymerase in 1X reaction buffer supplied with the polymerase (New England Biolabs). The first round PCR program involved 10 cycles of 96°C for 2 min, 55°C for 1 min, 72°C for 1 min and 25 cycles of 96°C for 1 min, 55°C for 1 min, 72°C for 1 min with a final extension at 72°C for 5 min. For the second round PCR of each individual locus, 0.1µl of the first amplification was used as template in a final volume of 25 µl containing 0.2 µM of each primer (internal forward primer and common reverse primer), 0.2 mM dNTPS, 2 mM $MgCl_2$ and 0.25U *Taq* Polymerase in 1X reaction buffer supplied with the polymerase (Bioline). The second round PCR program involved 35 cycles of 95°C for 30 sec, 56°C for 30 sec, and 72°C for 60 sec with a final extension at 72°C for 5 min. PCR products were separated on a 2% agarose gel by electrophoresis and stained with ethidium bromide.

The following primer sets were used for single cell genotyping. Referenced primer sets are indicated. The AlbCre-a primer set (internal forward primer and common reverse primer) was obtained from The Jackson Laboratories. All other primer sets were custom designed.

Locus	External Forward Primer (5'→3')	Internal Forward Primer (5'→3')	Common Reverse Primer (5'→3')	Product Size (bp)
Fah-a	TGAGAGGAGGGTA CTGGCAGCTAC	CCTGTGTTAAGGGG TCCTTG	TTGCCTCTGAACAT AATGCCAAC	174
Fah-b	CTAGGTCAATGGCT GTTTGG [1]	GGTGTTCCCTCTGC AGGA	GGACATACCAATTT GGCAAC [1]	115
R26R *lacZ*-a	GCACTTGCTCTCCC AAAGTC	AAAGTCGCTCTGAG TTGTTAT [2]	TCATCAAGGAAACC CTGGAC	~275
R26R *lacZ*-b	ACTATCCCGACCGC CTTACT	GTTTTGACCGCTGG GATCT	GCGATGCAATTTCC TCATTT	~450
Cre-a	CCGCAGAACCTGAA GATGTTC	GCGGTCTGGCAGT AAAAACTATC	GTGAAACAGCATTG CTGTCACTT	102
Cre-b	TTACGGCGCTAAGG ATGACT	TGGTCAGAGATACC TGGCCT	CTAATCGCCATCTT CCAGCAGG	196
Y-chrom (TSPY)-a	GGTGATAATTCCAC CCCTACTATG	TCCTTGGGCTCTTC ATTATTCTTAAC [3]	GAGAACCACGTTG GTTTGAGATG [3]	103
Y-chrom (TSPY)-b	CTGCCCTTTTGTAT GGGAAA	GTGGTCCCCTTTAG TACCAAC	CACATGCAGGCAG CATCTAT	200

REFERENCES

1. Grompe M, al-Dhalimy M, Finegold M, Ou CN, Burlingame T, et al. (1993) Loss of fumarylacetoacetate hydrolase is responsible for the neonatal hepatic dysfunction phenotype of lethal albino mice. Genes Dev 7: 2298-2307.

2. Soriano P (1999) Generalized lacZ expression with the ROSA26 Cre reporter strain. Nat Genet 21: 70-71.

3. Wang LJ, Chen YM, George D, Smets F, Sokal EM, et al. (2002) Engraftment assessment in human and mouse liver tissue after sex-mismatched liver cell transplantation by real-time quantitative PCR for Y chromosome sequences. Liver Transpl 8: 822-828.
